# Analysis of the Relationship between Alternative Respiration and Sterigmatocystin Formation in *Aspergillus nidulans*

**DOI:** 10.3390/toxins10040168

**Published:** 2018-04-20

**Authors:** Ákos P. Molnár, Zoltán Németh, Erzsébet Fekete, Michel Flipphi, Nancy P. Keller, Levente Karaffa

**Affiliations:** 1 Department of Biochemical Engineering, Faculty of Science and Technology, University of Debrecen, Egyetem tér 1, H-4032 Debrecen, Hungary; molnar.akos@science.unideb.hu (Á.P.M.); nemeth.zoltan@science.unideb.hu (Z.N.); drir.michelflipphi@gmail.com (M.F.); karaffa.levente@science.unideb.hu (L.K.); 2 Department of Medical Microbiology and Immunology, University of Wisconsin, Madison, WI 53706, USA; npkeller@wisc.edu; 3 Department of Bacteriology, University of Wisconsin, Madison, WI 53706, USA

**Keywords:** *Aspergillus nidulans*, alternative oxidase, respiration, light regulation, sterigmatocystin

## Abstract

*Aspergillus nidulans* has one gene for alternative oxidase (EC 1.10.3.11). To investigate the relationship between this mitochondrial terminal oxidase and the formation of the mycotoxin sterigmatocystin, the encoding *aodA* gene was both deleted and overexpressed. Relative to the wild-type, the cyanide-resistant fraction of respiration in the late stationary stage—when sterigmatocystin production occurs—doubled in the overexpressing mutant carrying three *aodA* gene copies, but decreased to 10% in the deletant. Essentially identical results were obtained regardless whether the cultures were illuminated or protected from light. In contrast, sterigmatocystin yield in the *aodA* deletant was about half of that in the control when grown in the dark, while *aodA* overexpression resulted in up to 70% more sterigmatocystin formed, the yield increasing with alternative oxidase activity. Results were quite different when cultures were illuminated: under those conditions, sterigmatocystin volumetric yields were considerably lower, and statistically unvarying, regardless of the presence, absence, or the copy number of *aodA*. We conclude that the copy number of *aodA*, and hence, the balance between alternative- and cytochrome C-mediated respiration, appears to correlate with sterigmatocystin production in *A. nidulans*, albeit only in the absence of light.

## 1. Introduction

**Figure 1:**
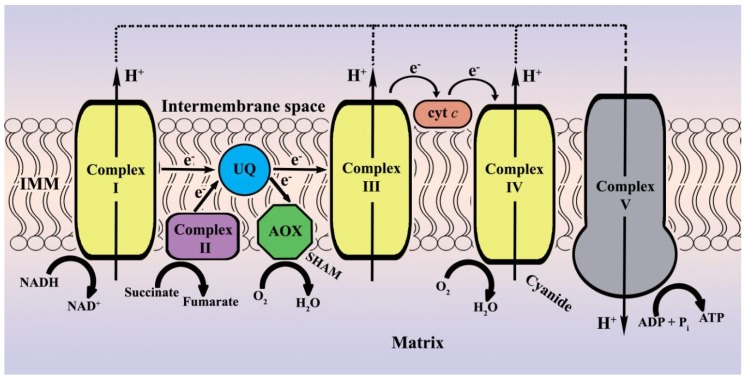
Schematic overview of cytochrome-mediated and alternative respiration.

Alternative oxidase (AOX) (systematic name: ubiquinol:oxygen oxidoreductase, nonelectrogenic; EC 1.10.3.11) occurs in many organisms: in plants and fungi, but also in animals and protists [1,2,3,4,5,6]. This cyanide-resistant terminal oxidase is located on the matrix side of the inner mitochondrial membrane (IMM). Unlike most of the cytochrome C oxidase (COX) subunits, alternative oxidase is encoded in the nuclear genome and it provides an “alternative” for the electron flow opposite to the canonical cytochrome-dependent pathway [4] (for a schematic overview of cytochrome-mediated and alternative respiration, see Figure 1). The branching point is at the level of coenzyme UQ (ubiquinone/ubiquinol), thus complexes III and IV of the electron transport system are bypassed. As a consequence, the alternative pathway after NADH: ubiquinone oxidoreductase (complex I), which oxidizes NADH to NAD+ and reduces ubiquinone to ubiquinol while translocating four protons, moves fewer protons across the inner mitochondrial membrane to generate a proton gradient, and thus provides less ATP by oxidative phosphorylation [7]. The energy of the four-electron oxidation of ubiquinol by oxygen to water is dissipated as heat. Additionally, the alternative respiration route is resistant to inhibitors of cytochrome complexes III and IV such as antimycin A, cyanide, or azide. AOX can be selectively blocked by aromatic hydroxamic acids such as salicyl hydroxamate (SHAM) [8]. AOX does not appear to perform a vital function; it is not present in yeasts such as *Saccharomyces cerevisiae* and *Schizosaccharomyces pombe*, nor in the complete vertebrata subphylum (including human). Although many ascomycetous filamentous fungi harbour two or even three homologue genes encoding an AOX in their genome, *Aspergillus nidulans*—a model system for biochemical and genetic research in multicellular fungi—features only a single alternative oxidase gene named *aodA* (GenBank accession number AB039832) [9,10].

Aflatoxins (AT) are among the most carcinogenic natural substances known to man. These mycotoxins belong to a large and diverse class of metabolites known as polyketides [11], and are produced by a variety of fungi, mainly from the genus *Aspergillus* [12]. These opportunistic fungi can contaminate cereal crops and other staple commodities before harvest or during storage, leading to economic losses and even famine in tropical countries [13]. Upon exposure, ATs can cause acute hepatic failure in humans and animals [14]. A less toxic fungal compound named sterigmatocystin (ST) is the penultimate intermediate in the biosynthesis of AT B_1_ [15,16]. However, in *A. nidulans*, ST is the stable end product of the corresponding secondary metabolism pathway [17], as it lacks the genes encoding the enzymes for the final two conversions (*aflP* and *aflQ*) from the AT gene cluster in *A. flavus* [18,19]. The biosynthesis of ST and AT shares similar regulatory mechanisms, including the two pathway-specific transcription factors AflR and AflJ (AflS), thus allowing the study of AT biosynthesis in *A. nidulans* without producing the potent mycotoxin itself [20,21]. The AT/ST biosynthetic pathway is well-characterized in *A. nidulans* [17,22], but many of the mechanisms by which environmental factors influence mycotoxin formation remain enigmatic [23,24]. Light—a fundamental environmental stimulus with broad effects—has been reported to cause diverse changes in AT/ST production [25,26,27,28], with data largely supporting an inhibitory impact of light on AT/ST biosynthesis through a conserved heteromeric complex known as the velvet complex [29].

Functionally, AOX has long been associated with metabolic conditions during which a switch from COX to AOX enables the cell to uncouple NADH reoxidization from ATP synthesis, allowing carbon catabolism to continue unabated [7,30]. This also occurs during secondary metabolite production, such as that of ST, under the prevalent low-energy conditions of the stationary growth phase. Here, we have studied possible relationships between the AOX activity and ST production of *A. nidulans* liquid (submerged, batch) cultures, employing well-controlled cultivation conditions conductive to ST synthesis in bioreactors. We found that this interrelation exists, but solely in the absence of light.

## 2. Results

### 2.1. Verification of the Experimental Strategy

To verify that *aodA* (locus AN2099) indeed encodes a physiologically relevant AOX in *A. nidulans*, we first compared the specific respiration rates of the deletion (Δ*aodA*) and OE mutants to a wild-type reference strain (RDIT 9.32). All cultures were in the rapid growth phase (at about 24 h after inoculation) at the time of sampling, when respiration is expected to be very high (Table 1). Specific overall respiration rates of the deletant (i.e., in the absence of any particular inhibitor) did not differ significantly from the reference. In contrast, OE mutants showed increased respiratory activity (*p* < 0.1), proportional to the *aodA* copy number (Table 1). In the presence of cyanide, the oxygen uptake rate of the wild-type reference dropped to about one-fifth, that is, the contribution of the cyanide-resistant fraction was some 20% of the total respiration. As expected, deletion of *aodA* has a profound effect on the cyanide-resistant oxygen uptake rate: values fell below 1 μM O_2_ min^−1^ gram^−1^ of dry cell weight (DCW), which is only 4% of the overall respiration rate. Respiration of the OE mutants, on the other hand, displayed a considerable increase (*p* < 0.1) of the cyanide-resistant fraction, which positively correlated with the *aodA* copy number. Statistically identical results were obtained with cultures grown in the dark and in the light, strongly suggesting that *aodA* expression does not appear to be influenced by (the absence or presence of) light.

To confirm that cyanide-resistant oxygen uptake mainly consists of AOX-driven respiration, we did experiments in the presence of the AOX-specific inhibitor SHAM. AOX-dependent respiration was calculated by subtracting the respiration simultaneously inhibited by cyanide and SHAM from cyanide-resistant respiration [31]. Irrespective of the cultivation condition, the so-called “residual respiration” always accounted for between 10% and 15% of the total cyanide-resistant respiration in the reference strain (data not shown). However, in the deletion strain, the addition of SHAM did not change the observed cyanide-resistant oxygen utilization, most likely due to enzymes that use molecular oxygen outside of the electron transport chain. *aodA* thus encodes the sole physiologically relevant SHAM-inhibited terminal oxidase in *A. nidulans*, and its activity can be reliably determined by monitoring oxygen uptake in the presence of cyanide. We therefore considered the experimental system appropriate for the purposes of this study.

### 2.2. Biomass Formation of *A. nidulans* Is Independent of the *aodA* Copy Number

**Figure 2:**
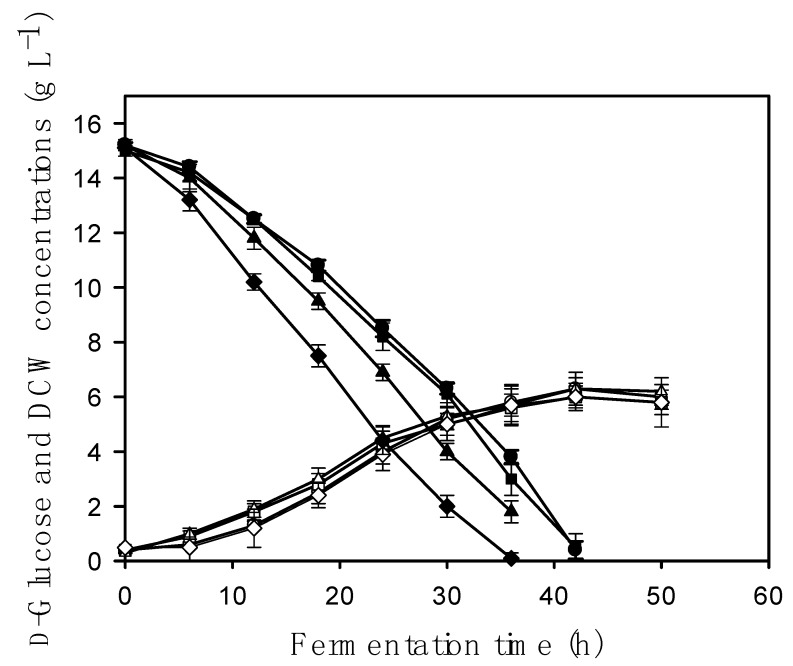
Time profiles of growth (white or open symbols) and residual d-glucose concentrations (black symbols) in submerged fermentations of *A. nidulans* grown in the dark in minimal media initially containing 15 g L^−1^ d-glucose as the sole carbon substrate. The wild-type strain is indicated by circles [⚫,Ο], the *aodA* deletant strain by squares [⬛,⬜], the *aodA* two-copy strain by triangles [▲,△], and the *aodA* three-copy strain by diamonds [◆,◇]. Mycelial inoculum grown from vegetative spores overnight on glycerol liquid medium and aseptically transferred into the bioreactors was used for all fermentations. Note that two variables share the same y-axis as they are expressed in the same unit definition: dry cell weight (DCW) and residual d-glucose concentration in the culture medium. For full details, see Materials and Methods.

**Figure 3:**
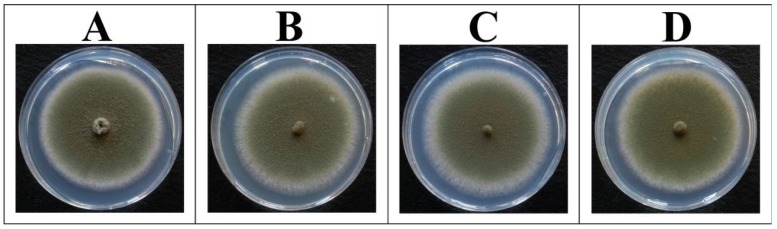
Point-inoculated (“single colony”) plate cultures of *A. nidulans* strains grown under continuous light on agar-solidified minimal medium with 15 g L^−1^ d-glucose as the sole source of carbon. (**A**) wild-type reference (RDIT 9.32); (**B**) *aodA* deletant (AMZN 3.37); (**C**) *aodA*^+^, two copies (AMZN 4.7); (**D**) *aodA*^+^, three copies (AMZN 5.13).

The finding that the oxygen uptake rates of the *aodA* OE mutants increased compared to the reference strain prompted us to test whether growth on glucose itself is correlated with the *aodA* copy number. Kinetic data from these submerged cultivations show that fungal biomass concentrations and concomitantly, specific growth rates were not statistically different at any of the time points tested (Figure 2 and Table 2). The same conclusion could be drawn from the colony diameters on point-inoculated plate cultures grown on agar-solidified medium under continuous light (Figure 3). Our data confirm an earlier report that an *aodA*-deleted strain grows as fast as a wild type strain [10]. By contrast, we found considerable differences (*p* < 0.1) in the residual d-glucose concentrations in the medium—which reflect d-glucose uptake rates (Table 2)—between the OE mutants and the reference or the *aodA* deletion strains (Figure 2). The copy number of the *aodA* gene in the OE mutants was positively correlated to the observed d-glucose uptake, that is, the cyanide-resistant alternative respiration and the d-glucose uptake rate increased in parallel. It should be noted, however, that residual d-glucose concentrations did not significantly differ between the wild-type reference and the *aodA* deletant, that is, the lack of alternative oxidase activity did not affect the d-glucose uptake rate (Table 2). Statistically identical results were obtained when cultures were illuminated or protected from light, suggesting that the growth of *A. nidulans*—at least under the conditions of this study—is not influenced by light.

### 2.3. Alternative Oxidase Activity in the Late Stationary Phase of Growth

In a previous publication [21], we demonstrated that ST formation only occurs at low specific growth rates. Consequently, in a medium with d-glucose as the growth substrate, ST production occurs only after carbon source exhaustion, during the late stationary phase of growth. Under the cultivation conditions used in this and our previous study, the maximal rate of ST formation occurred approximately 90 h after inoculation, while maximal ST concentrations were achieved at about 140 h of batch cultivation.

Respiration of the *A. nidulans* cultures with a different *aodA* copy number in the carbon-depleted, late stationary phase of the fermentations was in many ways quite different to that in the 24-h-old cultures (Table 1). In all four genetic backgrounds tested, total oxygen uptake rates considerably decreased (*p* < 0.1), but the extent of this change was dependent on the *aodA* copy number. The wild-type reference and the *aodA* deletion strains showed a reduction to 40% of their respective rates after 24 h of cultivation, while for the two OE strains, an even sharper decrease—to only 20% of the rates measured in the rapid growth stage—was recorded. While in the rapid growth stage, the overall oxygen uptake rate increased with the *aodA* copy number, in the late stationary phase (at 90 h), OE cultures exhibited lower respiration rates than the wild type. Importantly, the contribution of the cyanide-resistant component to the total respiratory activity of the OE strains in the late stationary growth phase increased only marginally from that in the rapid growth stage of the cultures, unlike what was observed in the wild-type control, where the contribution of alternative respiration clearly increased in the older mycelia. Nevertheless, the contribution of the cyanide-resistant fraction to the total respiration was dependent on the actual stage of growth: in all strains but the *aodA* deletants, the ratio of the cyanide-resistant respiration over the total respiration practically doubled in the lapse from the rapid growth stage (24 h) to the late stationary phase (90 h). At this late stage of the fermentations, up to 86% of the overall oxygen uptake of the three-copy OE strain was cyanide-resistant, while in the reference strain the ratio was 40%; the latter intriguingly similar to what was observed in the three-copy OE strain in the rapid growth phase. We conclude that while respiration rates had declined considerably by the late stationary phase of the cultures, the contribution of the cyanide-resistant terminal oxidase to the overall respiration markedly increased, again in direct relation with the *aodA* copy number.

### 2.4. Sterigmatocystin Formation in *A. nidulans* Is Dependent on the *aodA* Copy Number, but Only in the Absence of Light

**Figure 4:**
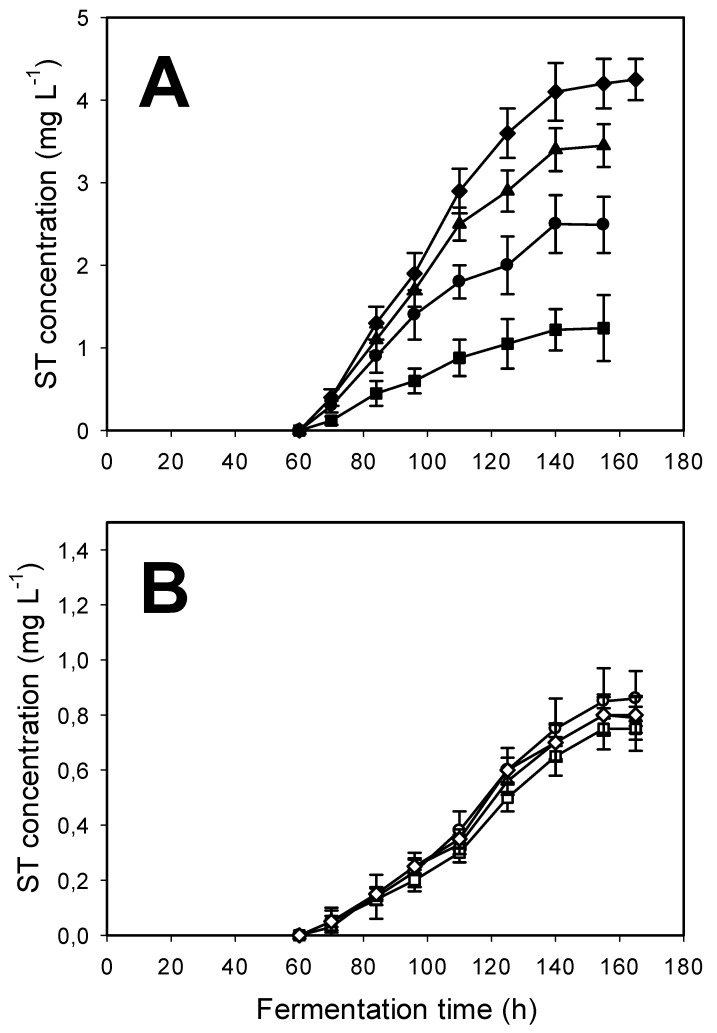
Time profile of sterigmatocystin (ST) production in submerged fermentations of *A. nidulans* grown in the dark (black symbols, Panel (**A**)) and in the light (white or open symbols, Panel (**B**)) in minimal media initially containing 15 g L^−1^ d-glucose as the sole carbon substrate. The wild-type reference strain is indicated by circles [⚫,Ο], the *aodA* deletant strain by squares [⬛,⬜], the *aodA* two-copy strain by triangles [▲,△], and the *aodA* three-copy strain by diamonds [◆,◇]. As all ST concentrations in the illuminated cultures are below 1 mg L^−1^, the scale of the y-axis is bigger in Panel (**B**) to illustrate that any observed differences are statistically irrelevant. For experimental details, see the legend to Figure 2 and the Section Materials and Methods.

**Figure 5:**
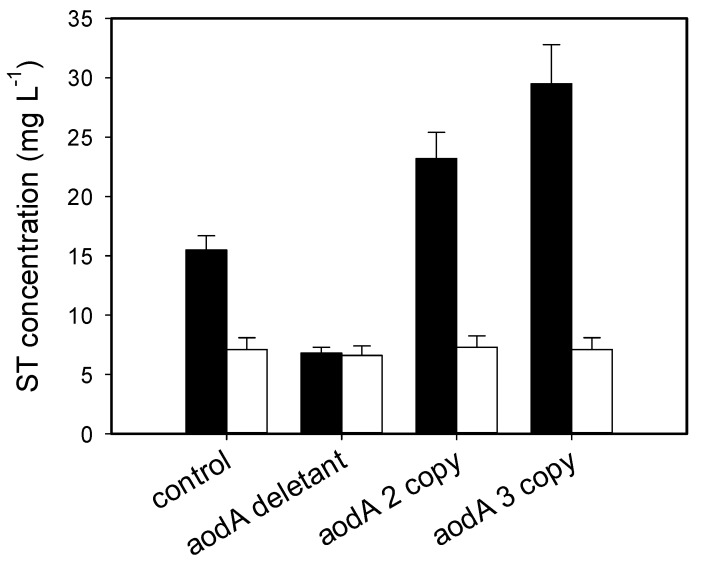
Sterigmatocystin production in point-inoculated (“single colony”) plate cultures of *A. nidulans* strains grown in the dark (black bars ⬛) or in continuous light (white or open bars ⬜) on agar-solidified minimal medium with 15 g L^−1^ d-glucose as the sole source of carbon. Samples were taken 5 days after inoculation, but statistically unvarying results were obtained from samples taken at day 7 (from a different set of plates; the latter data, not shown).

Kinetics of growth in our d-glucose minimal medium was similar in every *A. nidulans* strain investigated in this study, while d-glucose utilization rates as well as AOX activity—both early in the rapid growth stage and late in the carbon-depleted, stationary phase of cultures—were proportional to the *aodA* copy number, regardless of the presence or absence of light. To analyse the relationship between the aforementioned parameters and ST formation in *A. nidulans*, we monitored ST concentrations in the cultures (medium and biomass) (Figure 4). In qualitative terms, these time profiles were similar in that ST production did not occur before the complete depletion of d-glucose and the cessation of growth. Quantitatively, however, statistically significant differences were found. ST volumetric yield (mg L^−1^) of the *A. nidulans aodA* deletant strain was about half (50%) of that of the wild-type reference when grown in the dark. By contrast, *aodA* OE mutants featured a 50–70% increase in ST yield relative to the control strain, again in a copy-number-dependent manner. Importantly, results were quite different when cultures were continuously illuminated: ST volumetric yields of all cultures went down to about a third of what the control strain produced when grown in the dark, and were statistically unvarying, regardless of the presence, absence, or the copy number of *aodA*. This effect of light on ST production was confirmed upon testing point-inoculated plate cultures (Figure 5). Although production rates of ST in submerged, well-aerated cultures were significantly (up to five times) lower than on solidified medium, the trends were strikingly similar: in the dark, the *aodA* deletant yielded some 60% less, while the OE mutants produced considerably more (*p* < 0.1) ST compared to the wild-type control, in positive correlation with the number of functional *aodA* genes. In the light, no such correlation existed, and all the strains investigated in this study produced statistically nonvarying (*p* < 1) amounts of the mycotoxin that were approximately one-half of what the reference strain yielded on plates when grown in the dark. For the Δ*aodA* strain, that is, in the absence of alternative respiration, the ST yield on plates held in the dark was only marginally different from that accumulated when plates were subjected to continuous light.

## 3. Discussion

Many species of *Aspergillus*, including those from the section Flavi, such as *A. flavus* and *A. parasiticus*—prolific producers of AT—have more than one *aod* gene (our unpublished data). However, *A. nidulans* harbours a single AOX-encoding gene [9,10]. Since in our study, all copies of *aodA* were expressed from its genuine promoter, we could confirm that the copy number of *aodA* correlates well with the level of cyanide-resistant, SHAM-sensitive respiration in *A. nidulans*, making it an ideal experimental system to study the interplay between the formation of secondary metabolites and alternative respiration.

Recently, the negative consequences of a deregulated high overexpression of AOX on ST yield have been reported, using a nitrate-inducible promoter [32]. In a similar work in *Pichia pastoris*, it was concluded that the generally negative effects of deregulated, artificial AOX overexpression clearly indicate the importance of precise regulation of the encoding gene [33]. Moreover, nitrate is reported to inhibit the production of AT in many strains of *A. flavus* and *A. parasiticus* [26,34]. In *A. nidulans*—where one uses laboratory strains with the same, known ancestry instead of variants isolated from nature—it was found that a medium with ammonium was conductive to ST production in submerged cultures, while the same medium with nitrate was not [35]. Here, we have studied the consequences of a more subtle overexpression of the AOX gene from multiple copies under its own promoter for the production of the ST mycotoxin in a fully defined synthetic minimal medium with d-glucose as the sole carbon source and ammonium as the sole nitrogen source, under carefully controlled fermentation conditions.

Fermentation kinetics clearly showed that ST production started long after the external carbon source (d-glucose) had been depleted. Consequently, ST biosynthesis must be emanating from the breakdown of reserve carbon sources. A critical reappraisal of the existing literature suggested that reserve lipids play a prominent role in the biosynthesis of AT/ST [36]. Lipids within *Aspergillus*-contaminated seed increased the yield of mycotoxins in planta [23]. Defatted residues of vegetative origin yielded considerably less AT, but this effect could be reversed by the addition of vegetable oil to the defatted substrate [37]. Degradation of fatty acids into acetyl-CoA by beta-oxidation was shown to facilitate the formation of early ST intermediates in the peroxisomes in *A. nidulans* [38,39]. Indeed, there is a profound interconnection between fatty acids and polyketide secondary metabolites such as ST. Their biosynthesis shares acetyl-CoA and malonyl-CoA as the principal building blocks and NADPH as anabolic reducing equivalents, and the first step in ST biosynthesis—the formation of a hexanoyl-CoA condensation primer—is catalyzed by a fatty acid synthase (EC 2.3.1.85) [40]. In *A. versicolor*, fatty acids were formed during the rapid growth phase, and after exhaustion of the external carbon source, the fatty acid deposit was slowly consumed to maintain stationary growth concomitant with ST formation [41]. *A. versicolor*—a major source of ST contamination of food stock and animal feed—is more closely related to *A. nidulans* than species of the section Flavi, and the ST gene cluster is completely conserved between these two former species [42].

On the other hand, in the Sordariomycete *Acremonium chrysogenum*, AOX activity was heavily stimulated when the sugar-based growth medium was supplemented with plant oils, and this coincided with increased levels of cephalosporin C production [43]. In *Podospora anserina*, overexpression of AOX (from its own promoter) concurs with increased reserves of fatty acids and a decrease in 2-oxoglutarate concentrations, suggesting a major redirection of overall cellular metabolism [44]. Inhibition of complex III of the electron transport chain by antimycin A provoked increased transcription of the *P. anserina aodA* gene as a response to the respiratory stress, while the steady-state concentration of palmitate increased by almost two orders of magnitude. Thus, we speculate that AOX is accessory to the increased production of ST fueled by reserve lipids in the late stationary phase of growth, when the original external carbon source d-glucose is long depleted, but before the biomass starts to autolyze to mobilize cell wall polysaccharides.

By which mechanism(s) could AOX contribute to ST production? ST production predominantly takes place at the late stationary growth phase, when the growth rate is effectively zero, or even negative. ST synthesis requires considerable amounts of acetyl-CoA, ATP, and NADPH [15], so in the late stationary growth phase, this anabolic effort is serious. Recycling of the catabolic reducing equivalents (NADH) released during the mobilization of reserve carbon sources by COX would result in high ATP formation. However, in the absence of growth, mycelia do not require high(er) energy levels, and oxidative phosphorylation under such conditions inhibits further carbon catabolism. A switch from COX to AOX would enable the fungus to reoxidize NADH and maintain redox homeostasis without concomitant ATP production under low-energy-requiring conditions. Indeed, at the higher ST yields when mycelia were grown in the dark, ST production was positively correlated with AOX activity both in solid and submerged cultures.

Another aspect of the contribution of AOX to ST biosynthesis could be related to the formation of the anabolic reducing equivalents. NADPH is mostly generated by the oxidative part of the pentose phosphate pathway [45,46], which requires the continuous availability of glucose-6-phosphate, and this is facilitated by the increased flux through gluconeogenesis when the primary carbon source (d-glucose) is exhausted. In *A. nidulans*, mutations in the regulator genes *acuM* and *acuK* do not allow growth on acetate, nor on other C2 and C4 carbon sources that need the tricarboxylic acid (TCA) cycle to be catabolized [10]. Apart from phosphoenolpyruvate carboxykinase (AcuF) and fructose-1,6-bisphosphatase (AcuG), they—importantly—also control *aodA* and genes encoding mitochondrial carriers. These two zinc-cluster regulators have also been studied in *P. anserina*—known there as Rse2 and Rse3—where they are likewise responsible for the overexpression of AOX, the key enzymes of gluconeogenesis, as well as a NADH: ubiquinone oxidoreductase (EC 1.6.5.9) that allows bypassing of complex I of the electron transport chain [44,47]. Gluconeogenesis and AOX are thus controlled integrally by the same regulon that facilitates a shift of the metabolic flux towards fatty acid biosynthesis from the TCA cycle. Interestingly, fatty acid synthases are not regulated directly by the gluconeogenesis (Rse2/Rse3) regulon.

Another possibility may lie in the potential protection from reactive oxygen species (ROS). Pérez-Sanchez et al. [48] described that aeration increased solid-state lovastatin production by *A. terreus*, enhanced AOX activity, and lowered ROS accumulation. These authors hypothesized that AOX activity indirectly increased lovastatin production through a ROS-related mechanism. In contrast, it has been reported that oxidative stress, in the guise of an increased concentration of ROS, actually stimulates mycotoxin production in *Aspergilli*, while antioxidants in the medium reduce it [49,50,51,52,53]. While other work implies that both AT and its biosynthesis contribute to lowering the ROS levels [54], yet another study points to AT biosynthesis as a source of ROS [55]. In any case, AOX does not actively reduce ROS, nor is directly implicated in the production of antioxidant, but operates to maintain the redox balance while lowering the flux through the TCA cycle and the cytochrome electron transport chain such that less ROS will be produced in the mitochondria.

Irrespective of the mechanisms by which AOX activity stimulates ST synthesis, this incentive is clearly subordinate to antagonistic effects associated with the presence of light. The canonical theory is that the velvet transcription factor, on which ST synthesis strictly depends [25], is concentrated in the nucleus when the fungus grows in the darkness on a minimal d-glucose medium, but resides in the cytosol when the fungus grows in the light [56,57]. Illustrative of the regulatory complexity involved in mycotoxin production, it has also been reported that on rich media containing yeast extract, more ST is produced when mycelia are illuminated [25]. With regard to the effect of visual light on ST synthesis, the literature presents an interesting observation on the AT yields in a strain of *A. parasiticus* in a function of the temperature of the cultivation: Bennett et al. [58] obtained higher amounts in cultures incubated in the light at 20 °C and 25 °C than in those grown in the dark at those temperatures; however, at 30 °C, the AT yield was higher in cultures grown in darkness. Note that the default temperature to grow *A. nidulans* in the laboratory is 37 °C [59]. It has also been reported that the effect of light on ST biosynthesis as well as the targeting of velvet to the nucleus depends on the (initial) glucose concentration, at least when assessed on minimal medium plates [27]: At 1% (w/v), most ST was produced in the dark, but at 2% (w/v), the yield was higher in the light. The velvet protein was present in the nuclei both in the light and in the darkness at the tested glucose concentrations between 1 and 2%. In our experience, using the medium and growth conditions set out in Németh et al. [21], a velvet wild-type strain produced over twice as much ST when grown in the dark, regardless of whether the cultivation was on agar plates or in fermenters. Our results with the *aodA* mutant backgrounds imply that a basal level production under illumination (“stage 1”) is essentially unchanged regardless of the absence or presence of AOX, or its exact amount; that is, it seems irresponsive to the ratio of the alternative/total respiration. Again, this is true for mycelia grown on a solid surface and for biomass grown in submerged cultivation. Intriguingly, essentially the same levels of ST were detected in cultures of the Δ*aodA* deletion strain—devoid of alternative respiration—when cultivated protected from light, both in liquid and on plates. Our results further suggest that any increase in ST synthesis over the basal level (“stage 2”) can only be realized in the darkness (at 37 °C and 1.5% (w/v) initial glucose concentration) in genetic backgrounds that express AOX, that is, in strains that allow varying the ratio between alternative respiration and cytochrome-mediated respiration. We therefore speculate that only “stage 2” expression is mediated by the velvet complex, and that “stage 1” (basal level) expression may not require the velvet protein (to act) per se. Indeed, a gene (*rsmA*) has been identified of which overexpression remediates the lack of the *veA* gene for ST biosynthesis [60]. 

It is well documented that fungi break down AT/ST when the mycotoxin is added to a fungal culture, including those that do not produce AT/ST themselves [54,61,62]. However, we demonstrated that the rate of ST degradation by *A. nidulans* is much lower than that of production [21] under the conditions of this study. Moreover, ST production peaks at about 156–168 h after inoculation, regardless of the genetic background or the presence or absence of light. It thus seems unlikely that differences in ST degradation alone can explain our results, though we cannot rule out the possibility that different degrees of ST degradation are accessory to changes in de novo ST biosynthesis in the AOX-overexpressing backgrounds, when mycelia are grown protected from light.

## 4. Conclusions

In this work, the existence of a statistically supported, positive correlation between *aodA* copy number, AOX activity, and ST production was experimentally demonstrated. However, it was shown that this effect only occurs in the darkness; hence, an apparent inhibition of ST biosynthesis by visible light prevails over the stimulatory effects of AOX activity or the changed ratio between alternative and total respiration. The complexity of the regulation of AT/ST biosynthesis—which is influenced by integrated biochemical, environmental, and genetical factors—has thus far prevented the linkage of these observations to one defined operator system. Nevertheless, the current study emphasizes the importance of using well-controlled cultivation conditions, fully defined synthetic growth media, subtle gene overexpression, and strains with defined genetic backgrounds as opposed to natural isolates to investigate mycotoxin biosynthesis.

## 5. Materials and Methods

### 5.1. Fungal Strains, Media, and Culture Conditions

The *A. nidulans* strains used in this study are listed in Table S1. Throughout this study, all strains were maintained on Petri dishes containing Aspergillus Minimal Medium (AMM) solidified with 1.5% (w/v) agar, inoculated with vegetative spores. Supplements were added from sterile stock solutions where required.

AMM2 is standard Aspergillus Minimal Medium (AMM) [59] in which nitrate is substituted with 0.92 g diammonium tartrate L^−1^ while 0.1 g calcium chloride L^−1^ is also included. AMM2-based synthetic growth media used for submerged bioreactor cultivations—henceforth referred to as fermentations—were formulated and inoculated as described by [63]. Vitamins and other supplements were added from sterile stock solutions. d-Glucose was used at 1.5% (w/v) initial concentration. Agar-solidified complete medium was used to obtain vegetative spores and consisted of AMM2 supplemented with yeast extract (5 g L^−1^), peptone (10 g L^−1^), and corn steep liquor (10 g L^−1^). To quantitatively assess specific growth rates, biomass, and ST yields, fermentations were carried out in 2.5-L glass vessels (Sartorius AG, Göttingen, Germany) with a culture volume of 2 L, equipped with one six-blade Rushton disc turbine impeller. Operating conditions were pH 6.5, 37 °C, and 0.5 vvm (volumes of air per volume of liquid min^−1^). The dissolved oxygen (DO) level was maintained at 30% saturation and was controlled by means of the agitation rate; fungal AOX has a low affinity for molecular oxygen [64]. To minimize medium loss, the waste gas was cooled in a reflux condenser connected to an external cooling bath (4 °C) before exiting the system.

To investigate relations between alternative respiration and ST synthesis, well-known negative regulatory effects on ST formation by light [29] were taken into account; hence, fermentations—including inoculation and sampling—were performed either in the dark or in continuous white light (25 µE m^−2^ s^−1^), and the results were compared.

Shake flask cultures in 100 mL AMM2 with glycerol (1% v/v) as the carbon source in 500-mL glass Erlenmeyer flasks (VWR International Ltd., Debrecen, Hungary) grown for 24 h at 200 rpm and 37 °C in a rotary shaker (Infors AG, Bottmingen, Switzerland) were used as inoculum. These seed cultures were started with 10^6^ mL^−1^ freshly harvested *A. nidulans* conidiospores suspended in a sterile 1/10,000 Tween 20 solution, while 2-L bioreactors were inoculated with the biomass from 2 seed cultures. Thereto, the pregrown mycelia were harvested by filtration over a sintered glass funnel, thoroughly washed with cold sterile tap water, and transferred under aseptic conditions into the fermenter.

All chemicals were of analytical grade and were purchased from Sigma-Aldrich Kft. (Budapest, Hungary) unless stated otherwise.

### 5.2. Classical Genetic Techniques and Transformation

Conventional genetic techniques were employed to exchange markers by meiotic recombination [65]. Progeny of sexual crosses were tested for known auxotrophies using standard techniques.

### 5.3. Genomic DNA Isolation and Southern Blot Analysis

Mycelia were harvested by filtration over nylon mesh and thoroughly washed with sterile distilled water. Excess liquid was removed by squeezing the mycelia between paper sheets, and the biomass was rapidly frozen in liquid nitrogen. For nucleic acid isolation, frozen biomass was ground to a dry powder using a liquid nitrogen-chilled mortar and pestle. Genomic DNA was extracted using a NucleoSpin Plant II kit (Macherey-Nagel, Düren, Germany). Biomass sampling, genomic DNA isolation, DNA quality verification, quantification, and Southern blot analysis were all performed as described by [66].

### 5.4. Generation of Knockout Mutant Strains

The primers and plasmids used in strain generation are listed in Table S2. A gene deletion cassette was created in vitro with the double-joint PCR method [67] to delete the alternative oxidase (*aodA*) gene (locus AN2099). The cassette contained the *Aspergillus fumigatus* orotidine-5’-phosphate decarboxylase (*pyrG*) gene as primary selection marker. *A. nidulans* transformation was performed essentially as described by Tilburn et al. [68], using Glucanex (Novozymes; Copenhagen, Denmark) as the cell wall-lysing agent. Host strain TN02A3 was transformed with 10 µg of the linear deletion cassette. Uridine-prototroph transformants were tested for the absence of *aodA* by PCR using genomic DNA and specific oligonucleotide primers (Table S2). Transformants from which the *aod* gene was absent were purified twice to single cell colonies and maintained on selective minimal medium plates. 

### 5.5. Reintroduction of aodA into Gene-Deleted Backgrounds

The Δ*aodA* deletion mutant AMEF-001 (which has a *veA1*, *pyrA4*, and Δ*nkuA*-deleted background) was crossed with RJMP 155.55, a strain carrying the intact *nkuA* gene, resulting in AMZN 1.2. This strain harbours the wild-type *nkuA* gene and is auxotroph for pyridoxine and riboflavin. Strains with more copies of *aodA* (two or three; further on referred to as overexpressing [OE] mutants) were generated using a full-length gene fragment, PCR-amplified off genomic DNA (2836 bp, including a 905-bp long “promoter” (i.e., before the ATG) and an 836-bp long “terminator” (i.e., behind the STOP codon) sequence). Riboflavin auxotroph strain AMZN 1.2 was cotransformed with 10 µg of *aodA* fragment and 1 µg of pTN2 plasmid containing the *A. fumigatus riboB* gene [69] to yield riboflavin-prototroph primary transformants. These were PCR-verified for the presence of *A. fumigatus pyrG* and the *A. nidulans aodA* coding regions. The *aodA* copy number was subsequently estimated by Southern blot analysis in selected offspring (Supplementary Figure S1). The primers and plasmids used in strain generation are listed in Table S2.

Like almost all laboratory strains of *A. nidulans*, TN02A3 carries a missense mutation in the start codon of the *velvet* (*veA*) gene known as *veA1* [70]. This gene encodes a key regulator of sexual development and ST synthesis, and is involved in responses to light [25,29,71]. Strains lacking the *veA* gene are reportedly unable to produce ST under any conditions. For the current study, we generated strains that are wild type for *velvet* by meiotic recombination. The genetic background of the *aodA*-deleted (AMZN 1.2) and the thereof derived *aodA*-OE strains (AMZN 2.13 and AMZN 2.39, respectively) was equalised by sexual recombination, crossing each of them with RDIT 9.32, wild-type for *velvet*. Offspring was selected for which all known genotypic markers other than *aodA* (i.e., auxotrophies, spore color) were identical. These strains then were verified for the absence or presence of *aodA* and *velvet* by PCR, and the *veA+* strains that had acquired *aodA* in one or more copies or lacked this gene, were studied on plates for apparent growth phenotypes: strains AMZN 3.37 (Δ*aodA*), AMZN 4.7 (two copies of *aodA*), and AMZN 5.13 (three copies of *aodA*) were selected to be used in the fermentation experiments.

### 5.6. Analytical Methods

Dry cell weight (DCW) was determined from 10-mL culture aliquots as described by Fekete et al. [66]. The biomass was harvested on a preweighted glass wool filter and washed with cold tap water, after which the filter was dried at 80 °C until constant weight. Dry weight data reported in Results are the means of two separate measurements, which never deviated by more than 14%. Residual d-glucose concentrations were determined by HPLC with refractive index (RI) detection, as described by [63]. Biomass production rates (g_DCW_ h^−1^) were calculated from the increase in DCW over the time elapsed between two subsequent samplings (i.e., sampling time points); the highest of the thus obtained values was taken as the maximal specific growth rate of the culture. Likewise, glucose utilization rates (g/h) were calculated from the highest decrease in residual concentrations between two subsequent samplings.

The measurement of the mycelial respiration rates, including that of the alternative respiration, was performed with an oxygraphic electrode (Strathkelvin Instruments Ltd., North Lanarkshire, UK) at 37 °C, according to the manufacturer’s instructions. Potassium cyanide (1 mM) and salicyl hydroxamic acid (SHAM: 10 mM; [72]) were used to selectively inhibit the cytochrome C oxidase and the alternative oxidase, respectively. After these oxygen consumption measurements, the used biomass was harvested and DCW was determined, allowing calculation of the specific oxygen uptake rates.

Extraction of ST from the fungal culture (=medium plus biomass) and determination of its concentration was carried out as described by [21], using at least three technical replicates per experiment. The detection limit of the HPLC method is 0.1 mg L^−1^, which is well below the concentration range that *A. nidulans* can produce under submerged conditions, even in minimal medium. 

### 5.7. Reproducibility

All the data presented are means of at least three independent experiments (biological replicates). The data were analyzed and visualized by SIGMAPLOT (Jandel Scientific, San Jose, CA, USA), and standard deviations (SDs) were determined for each procedure. The SD values were always <14% of the mean values. The significance of changes in oxygen uptake, as well as in DCW, ST yield, and d-glucose concentrations in the growth medium of the alternative oxidase deletion mutant or the overexpressing (OE) strains, relative to the control cultures (RDIT 9.32), was assessed using the Student’s t-test with probability (*p*) values given in the Results section. 

## Figures and Tables

**Table 1:** Total and cyanide-resistant specific respiration rates as well as the percentage of the cyanide-resistant fraction within total respiration in the *Aspergillus nidulans* wild-type reference strain, the *aodA* deletant, and the two *aodA* overexpression mutants. Samples were taken after 24 h (rapid growth stage) and 90 h (late stationary phase) of cultivation under submerged conditions, in a minimal medium initially containing 15 g L^−1^ d-glucose as the sole carbon source.

	Time (h)	Wild-Type	*aodA* Deletant	*aodA*^+^ 2 Copies	*aodA*^+^ 3 Copies
**Total respiration** **(μM O_2_ min^−1^ g_DCW_^−1^)**	**24** **90**	20.7 ± 0.7 8.5 ± 0.3	19.4 ± 0.5 8.4 ± 0.5	25.4 ± 1.0 5.4 ± 0.2	30.6 ± 1.1 5.0 ± 0.1
**Cyanide-resistant respiration** **(μM O_2_ min^−1^ g_DCW_^−1^)**	**24** **90**	4.4 ± 0.2 3.4 ± 0.4	0.8 ± 0.1 0.4 ± 0.1	9.1 ± 0.3 3.9 ± 0.4	12.8 ± 0.5 4.3 ± 0.4
**Cyanide-resistant fraction (%)**	**24** **90**	21.2 40.0	4.1 4.7	35.8 72.2	41.8 86.0

**Table 2:** Derived kinetic parameters of the *A. nidulans* strains used in this study, grown in a minimal medium initially containing 15 g L^−1^ d-glucose as the sole carbon source.

Fungal Strain	Glucose Utilization Rate (g h^−1^)	Growth Rate (g_DCW_ h^−1^)
Reference (RDIT 9.32)	0.31± 0.03	0.15 ± 0.02
*aodA* deletant (AMZN 3.37)	0.29 ± 0.03	0.14 ± 0.02
*aodA*^+^, 2 copies (AMZN 4.7)	0.37 ± 0.03	0.15 ± 0.01
*aodA*^+^, 3 copies (AMZN 5.13)	0.44 ± 0.03	0.15 ± 0.02

## Supplementary Materials

The following are available online at http://www.mdpi.com/2072-6651/10/4/168/s1, Figure S1: Southern blot analysis of the *A. nidulans aodA* multi-copy strains, Table S1: *Aspergillus nidulans* strains used in this study, Table S2: Primers and plasmid used in this study.
