# Supplementary material for: Analysis of the Relationship between Alternative Respiration and Sterigmatocystin Formation in Aspergillus nidulans
Source: Toxins (Basel). 2018 Apr 20;10(4):168. doi: 10.3390/toxins10040168 (PMC5923334; doi:10.3390/toxins10040168)
Supplement: Supplementary file 1 [file toxins-10-00168-s001.pdf]

Supplementary Materials: Analysis of the Relationship between Alternative Respiration and Sterigmatocystin Formation in *Aspergillus nidulans*

Ákos P. Molnár, Zoltán Németh, Erzsébet Fekete, Michel Flipphi, Nancy P. Keller and Levente Karaffa

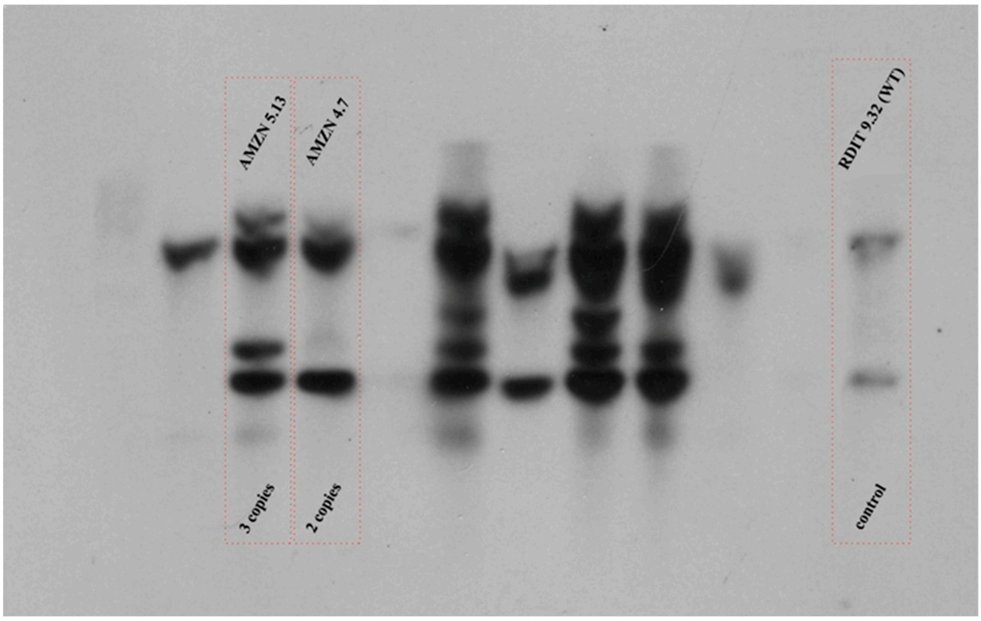

Figure S1: Southern blot analysis of the *A. nidulans aodA* multi-copy strains.

Table S1: *Aspergillus nidulans* strains used in this study.

Strain	Genotype	Reference
**RDIT 9.32 (FGSC A1252)**	*veA+; aodA*$^{+}$	**Tsitsigiannis et al. (2004)**
**TN02A3 (FGSC A1149)**	*veA1; pyroA4; pyrG89;* Δ*nkuA*	**Nayak et al. (2006)**
**RJMP 155.55**	*veA+; riboB2; wA3*	**Németh et al. (2016)**
**AMEF 001**	*veA1; pyroA4;* Δ*aodA;* Δ*nkuA*	**This study**
**AMZN 1.2**[1]	*veA+; riboB2; pyroA4;* Δ*aodA; wA3*	**This study**
**AMZN 2.13**	*veA+; pyroA4; aodA*$^{2+}$*; wA3*	**This study**
**AMZN 2.39**	*veA+; pyroA4; aodA*$^{3+}$*; wA3*	**This study**
**AMZN 3.37**[2]	*veA+;* Δ*aodA*	**This study**
**AMZN 4.7**[3]	*veA+; aodA*$^{2+}$	**This study**
**AMZN 5.13**[4]	*veA+; aodA*$^{3+}$	**This study**

All strains descended from FGSC A4, a genome sequenced strain.

All experiments described in the Results section were performed with the last 3 strains and the wild-type reference, RDIT 9.32.

Δ***aodA***: alternative oxidase negative; decreased cyanide-resistant respiratory rate.

aodA^{+}: alternative oxidase positive. $^{2+}$; $^{3+}$: number of ***aodA*** copies

[1] Offspring from a cross between AMEF 001 and RJMP 155.55.

[2] Offspring from a cross between AMZN 1.2 and RDIT 9.32.

[3] Offspring from a cross between AMZN 2.13 and RDIT 9.32.

[4] Offspring from a cross between AMZN 2.39 and RDIT 9.32.

Table S2: Primers and plasmid used in this study.

Primers		Sequence (5'-3')	Remark
veA+	Forward	TGTGTTATCCCATCAAGAGG	Han et al. (2010)
	Reverse	CTTGGCGCTGTAGACGATAA	
aodA	Forward	ATCCGCCCCTCGTCAAAAAAT	This study
	Reverse	TCAAACAACCTCCTCTCGT	
deletion cassette			This study
P1	Forward	AAAGTAGTCTCAGCGTAGCCT	*aodA*_5_flanking; for functional gene fragment (*aodA*)
P2	Reverse	CGGTTGAGCCGTTCAGGTACAGTACATGCAGGTAATGTTTCGCAATAGC	*aodA*_5_flanking
P3	Forward	TATGGTCCTGACATATCTGGTGGATCTACGAGAGGAGGTTGTTTGAG	*aodA*_3_flaking
P4	Reverse	AAAGATGAAAGGACAGGTGG	
P5	Forward	ATGTACTGTACCTGAACCG	*pyr4*
P6	Reverse	AGATCCACCAGATATGTCAG	
P7	Forward	TTTATTCTCGGCGTTTGTC	*aodA* nested
P8	Reverse	TAGAATAACAGCGGAAATGG	
P9	Reverse	CTCAACTAATAATCAATGCGC	for functional gene fragment (*aodA*)
aox_copyF	Forward	TAGTCTCAGCGTAGCCTCTTC	*aodA*; for Southern blot
aox_copyR	Reverse	GCGGATTGATTATTAGTTGAG	
Plasmid	Gene	Remark	
pTN2	*riboB2*	Nayak et. al (2006)	
